# Network Pharmacology-Based Strategy to Investigate the Pharmacologic Mechanisms of *Atractylodes macrocephala* Koidz. for the Treatment of Chronic Gastritis

**DOI:** 10.3389/fphar.2019.01629

**Published:** 2020-01-29

**Authors:** Songhong Yang, Jinlian Zhang, Yiqi Yan, Ming Yang, Chao Li, Junmao Li, Lingyun Zhong, Qianfeng Gong, Huan Yu

**Affiliations:** ^1^ School of Pharmacy, Jiangxi University of Traditional Chinese Medicine, Nanchang, China; ^2^ Chinese Medicine Research Institute, Tianjin University of Traditional Chinese Medicine, Tianjin, China

**Keywords:** network pharmacology, *Atractylodes macrocephala* Koidz., chronic gastritis, bioactive ingredients, mechanism of action

## Abstract

Chronic gastritis (CG) is an inflammatory disease. *Atractylodes macrocephala* Koidz (AMK) is employed in traditional Chinese medicine (TCM) to treat various disorders. AMK can be efficacious against CG, but the active ingredients, drug targets, and its exact molecular mechanism are not known. We employed network pharmacology to analyze the active ingredients, drug targets, and key pathways of AMK in CG treatment. Seventy-seven AMK candidate ingredients were selected from four databases, and 27 active ingredients were selected for CG treatment. Twenty-five overlapping gene symbols related to CG and drugs were obtained from GeneCards and OMIM databases. A protein–protein interaction (PPI) network and TCM comprehensive network (Drug–Ingredients–Gene symbols–Disease network) were constructed, and 528 Gene Ontology (GO) terms and 26 pathways were obtained by analyses of enrichment of GO pathways and Kyoto Encyclopedia of Genes and Genomes (KEGG) pathways. We suggest that the interleukin-17 signaling pathway, C-type lectin receptor signaling pathway, tumor necrosis factor signaling pathway, and AGE-RAGE signaling pathway in diabetic complications might serve as the key points and principal pathways for CG treatment. We also evaluated the reliability of some important active ingredients and targets by *in vitro* experiments. We showed that AMK probably influences the inflammatory response, amino acid synthesis, and energy metabolism when treating CG. This study provides novel insights for researchers to explore the mechanism of action of TCM systematically.

## Introduction

Chronic gastritis (CG) is an inflammatory disease in which the epithelium of the gastric mucosa is invaded by various pathogenic factors, which results in persistent and chronic inflammatory changes. CG can be divided into three categories: chronic non-atrophic, chronic atrophic, and “special.” If not treated in a timely manner, CG can transform into gastric cancer (Ohata et al., 2004; Yue et al., 2018; Osaki et al., 2019; Han et al., 2019). Most patients with CG do not have obvious symptoms, and the main symptom is dyspepsia, which is nonspecific. Some CG patients can present with abdominal pain, bloating, and other symptoms of indigestion.

Traditional Chinese medicine (TCM) has been used to treat various diseases for thousands of years. In TCM theory, CG is divided into six types according to the pattern of: accumulation of damp heat in the spleen–stomach; dampness obstructing the spleen–stomach; spleen–stomach Qi deficiency; spleen–stomach deficiency cold; liver–Qi stagnation; stagnant heat in the liver–stomach (Zhu et al., 2014).

*Atractylodes macrocephala* Koidz. (AMK) can invigorate the spleen and Qi, reduce dampness and moisture, act as an antiperspirant, and stop fever developing. In TCM, it is often used to treat spleen deficiency, malnutrition, abdominal distension, diarrhea, dizziness, palpitations, edema, spontaneous sweating, and fetal restlessness (The State Commission of Chinese Pharmacopoeia, 2010; Tang et al., 2012; Zhao, 2013).

In recent years, network pharmacology has been used widely in TCM research. By using “web”-based approaches, network pharmacology can systematically determine the effects and mechanisms of drugs used to treat complex diseases at molecular, cellular, tissue, and biologic levels.

Danlu capsules, the Maxing Ganshi Decoction, Wei Pi Xiao Decoction and Xiaoyao powder have been adopted widely in TCM (Liu et al., 2016; Huang et al., 2017; Song et al., 2018; Yang et al., 2019). AMK can be efficacious for CG treatment, but the active ingredients, drug targets, and the exact molecular mechanism are not known.

**Figure 1:**
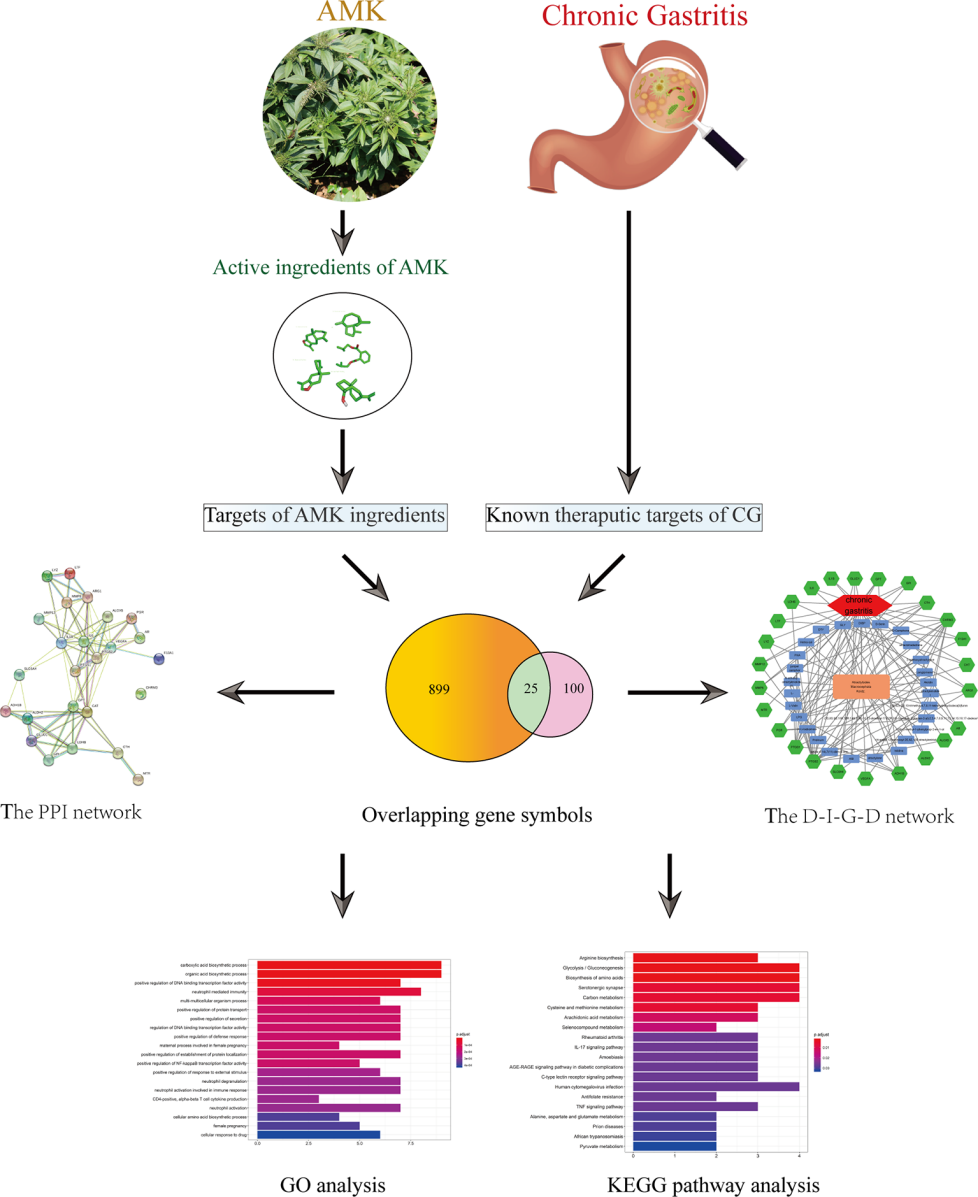
Flowchart of a network pharmacology-based strategy to investigate the pharmacologic mechanisms of *Atractylodes macrocephala* Koidz. for treatment of chronic gastritis.

We used network pharmacology to analyze the active ingredients, drug targets, and key pathways of AMK in CG treatment (**Figure 1**). Results suggested that AMK may have a therapeutic effect against CG because it can regulate key factors in the inflammatory pathway.

## Materials and Methods

### Screening of Active Ingredients

All ingredients contained in AMK were searched for in the Traditional Chinese Medicine Systems Pharmacology Database and Analysis Platform (TCMSP; http://lsp.nwu.edu.cn/tcmsp.php), Chinese Academy of Sciences Chemistry Database (www.organchem.csdb.cn), TCM Database@taiwan (http://tcm.cmu.edu.tw), and Traditional Chinese Medicine Integrated Database (https://omictools.com/tcmid-too). TCMSP is a special platform based on systems pharmacology for Chinese herbal medicines that includes the relationships between drugs, targets, and diseases. TCMSP has information on 500 drugs and >12,000 ingredients (Ru et al., 2014).

“Oral bioavailability” (OB) refers to the percentage of unmodified drugs that enters the circulatory system after oral administration (Xu et al., 2012; Liu et al., 2013). OB is also an important indicator for objective evaluation of the internal quality of drugs (Alam et al., 2014). The higher the OB of an ingredient, the higher is the likelihood that it can be used clinically. “Drug likeness” (DL) is a vague concept that refers to the similarity between ingredients and known drugs (Walters and Murcko, 2002; Tao et al., 2013). Ingredients with DL properties are not drugs, but can become drugs. This class of ingredient comprises drug-like molecules or drug analogs. The Tanimoto coefficient was used to evaluate the DL index of the molecules in AMK using the following formula:

(1) $$T(\text{α},\text{β})=\frac{\text{α}\times \text{β}}{{\text{α}}^{2}+{\text{β}}^{2}-\text{α}\times \text{β}}$$

where α is the molecular property of the AMK ingredient on the basis of Dragon software (www.talete.mi.it/products/dragon_description.htm) and β denotes the average molecular property for all drugs in the DrugBank database (www.drugbank.ca/) (Mauri et al., 2006).

Most ingredients in Chinese formulations have poor pharmacologic properties, so they cannot bind to specific protein targets on cells efficaciously. Therefore, several researchers have recommended that molecules with OB ≥ 30% or DL ≥ 0.18 are considered to have better pharmacologic effects, and are selected as candidate ingredients for further analyses (Liu et al., 2016; Huang et al., 2017; Yang et al., 2019). We used this principle to screen for active ingredients to be used in further analyses.

### Identification of the Related Targets and Gene Symbols of AMK Ingredients

Target forecasts and target-prediction methods can be divided into four categories based on: the predictions of a ligand (chemical similarity and pharmacophore model); the predictions of a receptor (molecular docking); machine learning (the molecules in a database must have a clear correspondence with the target, and the name of the target must be standardized); a combined forecast. Considering the limitations of the experimental conditions of molecular docking and machine learning and our previous experience, we chose a prediction method based on ligands for subsequent research (Yu et al., 2012; Wu et al., 2016; Wang et al., 2017).

All the protein targets of the ingredients in AMK were retrieved from TCMSP (http://lsp.nwu.edu.cn/tcmsp.php). We removed redundant information and retained only those targets that could interact directly with each of these ingredients in AMK. Then, the target was transformed using the UniProt knowledge database (www.uniprot.org) with the selected species as *Homo sapiens*. After deletion of redundant items, data were merged to obtain gene symbols.

### Acquisition of Gene Targets for CG

We collected gene targets for CG from two sources. The first source was the GeneCards v4.9.0 (www.genecards.org/). GeneCards is a searchable, comprehensive database that offers all comments and predicts human genetic information comprehensively in a user-friendly manner (Rebhan, 1997; Safran et al., 2010). We used the keyword “chronic gastritis” to search this database. The second source was the Online Mendelian Inheritance in Man (OMIM) database (www.omim.org/, updated on 28 February 2019) (Hamosh et al., 2005).

### Construction of a Drug–Ingredients–Gene Symbols–Disease (D–I–G–D) Network

First, we intersected the obtained drug targets with the genes associated with a disease and obtained a Venn diagram of the intersected gene symbols. Then, we built a network of complex information based on interactions between the drug (AMK), ingredients, gene symbols, and disease (CG). Next, we used Cytoscape v3.7.1 (www.cytoscape.org/) (Shannon, 2003; Su et al., 2014) to undertake visual analyses of the D–I–G–D network (which is a graphical display of network analyses and editing software).

### Construction of a Protein–Protein Interaction (PPI) Network

PPI data were obtained from the String v11.0) https://string-db.org/, updated on 19 January 2019) (Hsia et al., 2015). Then, the target was transformed using the UniProt knowledge database. After deletion of redundant items, data were merged to obtain gene symbols. Finally, we searched these gene symbols using the “multiple proteins” option while simultaneously setting the organism to *Homo sapiens*. Then, the PPI network of AMK active ingredients–target symbols and chronic gastritis-related target symbols was constructed.

### Enrichment of Gene Ontology (GO) Pathways

GO is an international standard classification system for gene function (Ashburner et al., 2000). We used Bioconductor (R) v3.8 (http://bioconductor.org/, released on 31 October 2018) for analyses.

### Enrichment of the Kyoto Encyclopedia of Genes and Genomes (KEGG) Pathway

The KEGG database analyzes the metabolic pathways of gene products in cells and the functions of these gene products (Kanehisa and Susumo, 2000). We used Bioconductor (R) v3.8 (http://bioconductor.org/, released on 31 October 2018) for analyses.

### Computational Validation of Ingredients–Targets Interactions

We wished to ascertain the interaction between active ingredients and their protein targets and explore their binding modes. Hence, we selected three active ingredients and three targets, a total of four ingredients–targets interactions for verification of molecular docking. We used GOLD v5.1 (a genetic algorithm-based docking program to dock protein–ligand complexes). We obtained the X-ray crystal structures of interleukin (IL)-6, IL-1β, and muscarinic acetylcholine receptor M3 (CHRM3) from the RCSB Protein Data Bank (PDB) (www.rcsb.org); the PDB entry code for these proteins is 1ALU, 2NVH, and 4DAJ, respectively. During molecular docking, we adopted the GOLD Score fitness function. The GOLD fitness function comprised three main terms: a hydrogen energy; a pairwise van der Waals steric energy between the protein and the ligand; and an internal energy term for the ligand.

### Experimental Validation

#### Reagents

Atractylenolide-I (ATL-I; purity ≥98%) was purchased from Must Biotechnology (Chengdu, China). A stock solution of 100 mM ATL-I in dimethyl sulfoxide was prepared and stored at 4°C. Enzyme-linked immunosorbent assay (ELISA) kits for IL-6 and IL-1β were purchased from Elabscience Biotechnology (Wuhan, China).

#### Cell Culture

RAW264.7 cells were obtained from the cell bank of the Chinese Academy of Sciences (Shanghai, China). RAW264.7 cells were cultured in Dulbecco’s modified Eagle’s medium (Gibco, Billings, MT, USA) with 10% fetal bovine serum (Gibco). Cells were cultured at 37°C in an atmosphere of 5% CO_2_ for all experiments.

#### Assay to Measure Cell Viability

RAW264.7 cells in the logarithmic phase were seeded at 8 × 10^3^ cells/well in 96-well culture plates. After incubation for 24 h, Raw264.7 cells were exposed to ATL-I (0, 20, 40, 60, 80, or 100 μmol/L). After treatment for 24 h, 20 μL of Cell Counting Kit (CCK-8) assay solution (Biosharp, Hefei, China) was added to each well, and cells were incubated for 4 h at 37°C in an atmosphere of 5% CO_2_. The absorbance at 450 nm was measured by a microplate reader. Cell survival was calculated as: absorbance/absorbance of control ×100%.

#### Determination of Levels of IL-6 and IL-1β by ELISAs

RAW264.7 cells (5 × 10^3^ cells/well) were incubated with lipopolysaccharide (LPS; 1 μg/mL) for 24 h and then treated with ATL-I (20, 40, or 60 μM) for 24 h. Supernatants were harvested and the level of IL-6 and IL-1β determined by ELISA kits (Biosharp).

#### Real-Time Quantitative Polymerase Chain Reaction (qRT-PCR)

Total RNA was extracted with TRIzol^®^ Reagent (Thermo Scientific, Waltham, MA, USA), and reverse-transcribed with oligo-DT using HiScript™ Reverse Transcriptase (Vazyme, Beijing, China) according to manufacturer instructions. The primers used were synthesized by Tsingke (Beijing, China). The sequences were (forward and reverse, respectively) 5′-TCAGGCAGGCAGTATCACTC-3′ and 5′-AGCTCATATGGGTCCGACAG-3′ for IL-1β; 5′-CACAGAGGATACCACTCCCAACAGA-3′ and 5′-ACAATCAGAATTGCCATTGCACAAC-3′ for IL-6; 5′-ATGGGTGTGAACCACGAGA-3′ and 5′-CAGGGATGATGTTCTGGGCA-3′ for the internal control glyceraldehyde 3-phosphate dehydrogenase (GAPDH).

qRT-PCR was done using SYBR™ Green Master Mix (Vazyme) in the QuantStudio 6 Flex system (Applied Biosystems, Foster City, CA, USA). The PCR cycling profile was: one cycle at 50°C for 2 min and 95°C for 10 min, 40 cycles at 95°C and 60°C for 30s. Fluorescence signals were detected using the QuantStudio 6 Flex system. Gene-expression data were normalized to that of the endogenous control GAPDH. The 2^−ΔΔCT^ method was the basis for relative expression of genes.

### Statistical Analyses

Data are the mean ± SD. The significance of results was determined based on one-way analysis of variance using Prism 8.0.1 (Graphpad, San Diego, CA, USA). *p* < 0.05 was considered significant. All experiments were repeated at least three times.

## Results

### Screening of Active Ingredients

Seventy-seven AMK candidate ingredients were selected from three databases (**Supplementary Table S1**). To identify the active ingredients of AMK, two classical absorption, distribution, metabolism, and excretion (ADME) parameters, OB and DL, were used for screening. Some ingredients were not in accordance with the standard of screening ingredients and were also likely to produce therapeutic effects in the human body. To study this issue more comprehensively, although they did not meet the screening criteria, these candidate ingredients were retained as active ingredients in our study. For example, ATL-I had a low DL, but it was retained as an active ingredient because it is the major constituent of AMK (Zhang et al., 1998; Li et al., 2007). We found that proliferation of human gastric cancer (mgc-803) cells was inhibited by ATL-I in a time-dependent and dose-dependent manner (*p* < 0.05). ATL-I inhibited expression of Notch-1, Hey-1, Jagged-1, and Hes-1 in the Notch signaling pathway. ATL-I inhibited expression of the mRNA of Notch-1, Hey-1, Jagged-1, and Hes-1 (*p* < 0.05). ATL-I has been shown to inhibit proliferation of mgc-803 cells in human gastric cancer by inhibiting the Notch signaling pathway (Ma et al., 2014). ATL-I could inhibit the increase in vascular permeability caused by acetic acid and can resist proliferation of granuloma tissue. AMK also has a therapeutic effect on acute or chronic inflammation (Chen, 1987; Li et al., 2007). It is important to note that although the pharmacokinetic values of several ingredients were relatively low, they had biologic activity, so they were also regarded as candidate ingredients. In addition, we expanded the criteria for screening beyond the ADME principle. We postulated that, as long as the candidate ingredients in AMK intersect with the targets of CG, they can be considered to be active ingredients.

In summary, 27 ingredients were selected as active ingredients in AMK (**Table 1**).

**Table 1:** A total of twenty-seven ingredients were selected as the details of the active ingredients of AMK in this study.

No.	Mol ID	CAS No.	Molecule Name	OB	DL	Concentration： DW (mg/g) or The relative content = Volatile ingredient / total volatile oil×100%	Reference
1	MOL000050	56-40-6	Glycine	48.74	0.00	0.70 mg/g	C.-Y. Hu et al. “Nutritional components of wild plant Qibaizhu (Atractylodes macracephala Koidz),” Journal of Biology, 3 (2005).
2	MOL000054	74-79-3	Arginin	47.64	0.03	16.1 mg/g	C.-Y. Hu et al. “Nutritional components of wild plant Qibaizhu (Atractylodes macracephala Koidz),” Journal of Biology, 3 (2005).
3	MOL000056	60-18-4	L-Tyrosine	57.55	0.05	1.60 mg/g	C.-Y. Hu et al. “Nutritional components of wild plant Qibaizhu (Atractylodes macracephala Koidz),” Journal of Biology, 3 (2005).
4	MOL000061	147-85-3	L-Proline	77.57	0.01	2.30 mg/g	C.-Y. Hu et al. “Nutritional components of wild plant Qibaizhu (Atractylodes macracephala Koidz),” Journal of Biology, 3 (2005).
5	MOL000064	302-84-1	DL-Serine	83.59	0.01	1.80 mg/g	C.-Y. Hu et al. “Nutritional components of wild plant Qibaizhu (Atractylodes macracephala Koidz),” Journal of Biology, 3 (2005).
6	MOL000065	6899-03-2	L-aspartic acid	79.74	0.02	5.30 mg/g	C.-Y. Hu et al. “Nutritional components of wild plant Qibaizhu (Atractylodes macracephala Koidz),” Journal of Biology, 3 (2005).
7	MOL000067	7004-03-7	Valine	53.33	0.01	3.60 mg/g	C.-Y. Hu et al. “Nutritional components of wild plant Qibaizhu (Atractylodes macracephala Koidz),” Journal of Biology, 3 (2005).
8	MOL000071	30641-68-0	2-amino-3-(3H-imidazol-4-yl)propanoic acid	53.18	0.03	0.40 mg/g	C.-Y. Hu et al. “Nutritional components of wild plant Qibaizhu (Atractylodes macracephala Koidz),” Journal of Biology, 3 (2005).
9	MOL000042	115967-49-2	(2S)-2-aminopropanoic acid	87.69	0.01	1.60 mg/g	C.-Y. Hu et al. “Nutritional components of wild plant Qibaizhu (Atractylodes macracephala Koidz),” Journal of Biology, 3 (2005).
10	MOL000041	5297-02-9	(2S)-2-amino-3-phenylpropanoic acid	41.62	0.04	0.70 mg/g	C.-Y. Hu et al. “Nutritional components of wild plant Qibaizhu (Atractylodes macracephala Koidz),” Journal of Biology, 3 (2005).
11	MOL000047	473-04-1	juniper camphor	33.30	0.10	0.66 %	LI, Ying. GC-MS analysis of the essential oil components in Atractylodis Macrocephalae Rhizoma and its stir-baked product. Chinese Journal of Pharmaceutical Analysis 33.7 (2013): 1210-1217.
12	MOL000038	260-94-6	Akridin	33.71	0.10	0.31 %	Q. Qiu,et al. Study on the chemical constituents of the volatile oil of atractylodes atractylodes by gc-ms. Chinese Traditional and Herbal Drugs. vol.11, pp.23-24+44, 2002.
13	MOL000025	1493692	α-Longipinene	53.26	0.12	0.15 %	LI, Ying. GC-MS analysis of the essential oil components in Atractylodis Macrocephalae Rhizoma and its stir-baked product. Chinese Journal of Pharmaceutical Analysis 33.7 (2013): 1210-1217.
14	MOL000019	5794-03-6	D-Camphene	34.98	0.04	0.02 %	Zhou J.J., Xie G.R., Yan X.J. Chemical composition of traditional Chinese medicine. Science Press, 2009
15	MOL000018	124-76-5	DL-Isoborneol	86.98	0.05	6.71 %	X. D, Li. et al. Extraction process of the essential oil from Zingiber officinale Rosc. and Atractylodes macrocephala Koidz. Journal of Shenyang Pharmaceutical University 1 (2003).
16	MOL000022	113269-36-6	14-acetyl-12-senecioyl-2E,8Z,10E-atractylentriol	63.37	0.3	Identified	Yao, C.M., Yang, X.W. Bioactivity-guided isolation of polyacetylenes with inhibitory activity against NO production in LPS-activated RAW264.7 macrophages from the rhizomes of Atractylodes macrocephala. Ethnopharmacol. 151, pp. 791–799, 2014.
17	MOL000030	103729-80-2	(±)-2-methyl-1-phenylprop-2-en-1-ol	75.1	0.03	Identified	Zhou J.J., Xie G.R., Yan X.J. Chemical composition of traditional Chinese medicine. Science Press, 2009
18	MOL000072	113269-35-5	8β-ethoxy atractylenolide III	35.95	0.21	Identified	Z.-L. Chen, "The acetylenes from Atractylodes macrocephala," Planta Medica, 53, 5, pp. 493–494, 1987.
19	MOL000048	19912-61-9	furanodiene	43.17	0.1	0.314 %	Q. Zhang, Z. W. Li. Studies on chemical constituents of the essential of Atractylodes macrocephala Koidz. West china journal of pharmaceutical sciences.1997,2.
20	MOL000023	5989-27-5	Limonene	39.84	0.02	0.007 %	Zheng J. Analysis on Chemical Constituents of Essential Oils from Different Varieties of Atractylodes macrocephala by GC-MS. China Pharmacy 31 (2007).
21	MOL000033	64997-52-0	Beta-sitosterol	36.23	0.78	Identified	Sun, Xue, et al. “Influence of sulfur fumigation on the chemical profiles of Atractylodes macrocephala Koidz. evaluated by UFLC–QTOF–MS combined with multivariate statistical analysis.” Journal of pharmaceutical and biomedical analysis 141 (2017): 19-31.
22	MOL000066	25246-27-9	Alloaromadendrene	53.46	0.1	0.039 %	Zheng J. Analysis on Chemical Constituents of Essential Oils from Different Varieties of Atractylodes macrocephala by GC-MS. China Pharmacy 31 (2007).
23	MOL000057	84-69-5	DIBP	49.63	0.13	Identified	Zhou J.J., Xie G.R., Yan X.J. Chemical composition of traditional Chinese medicine. Science Press, 2009
24	MOL000060	54707-47-0	selina-4(14),7(11)-dien-8-one	32.31	0.1	Identified	Sun, Xue, et al. “Influence of sulfur fumigation on the chemical profiles of Atractylodes macrocephala Koidz. evaluated by UFLC–QTOF–MS combined with multivariate statistical analysis.” Journal of pharmaceutical and biomedical analysis 141 (2017): 19-31.
25	MOL000043	73069-13-3	atractylenolide I	37.37	0.15	0.375 mg/g	Y.-H. Meng et al. “Determination of Atractylone and other four effective components in Atractylodes macrocephala and its processed products by HPLC,” Chemical Engineer, 33, 08 pp.24-26, 2019.
26	MOL000046	6989-21-5	atractylone	41.1	0.13	46.05 %	LI, Ying. “GC-MS analysis of the essential oil components in Atractylodis Macrocephalae Rhizoma and its stir-baked product,” Chinese Journal of Pharmaceutical Analysis, vol.33 no.7 pp. 1210-1217, 2013.
27	MOL000049	61206-10-8	3β-acetoxyatractylone	54.07	0.22	Identified	Shan, Guo-Shun, et al. “Metabolomic study of raw and processed Atractylodes macrocephala Koidz by LC–MS.” *Journal of pharmaceutical and biomedical analysis* 98 (2014): 74-84.

### Identification of the Related Targets and Gene Symbols of the Ingredients in AMK

After collection from the TCMSP database, conversion into the UniProt database, and deletion of redundant items, 27 ingredients in AMK and 100 known target symbols related to them were obtained (**Supplementary Table S2**).

### Acquisition of Known Therapeutic Gene Targets for CG

A total of 901 known therapeutic target symbols for CG were collected from the GeneCards database. In addition, 23 known therapeutic targets for CG were obtained from the OMIM database. After elimination of redundancies, 899 known therapeutic targets for CG were collected (**Supplementary Table S3**).

### Analyses of the D-I-G-D Network

**Figure 2:**
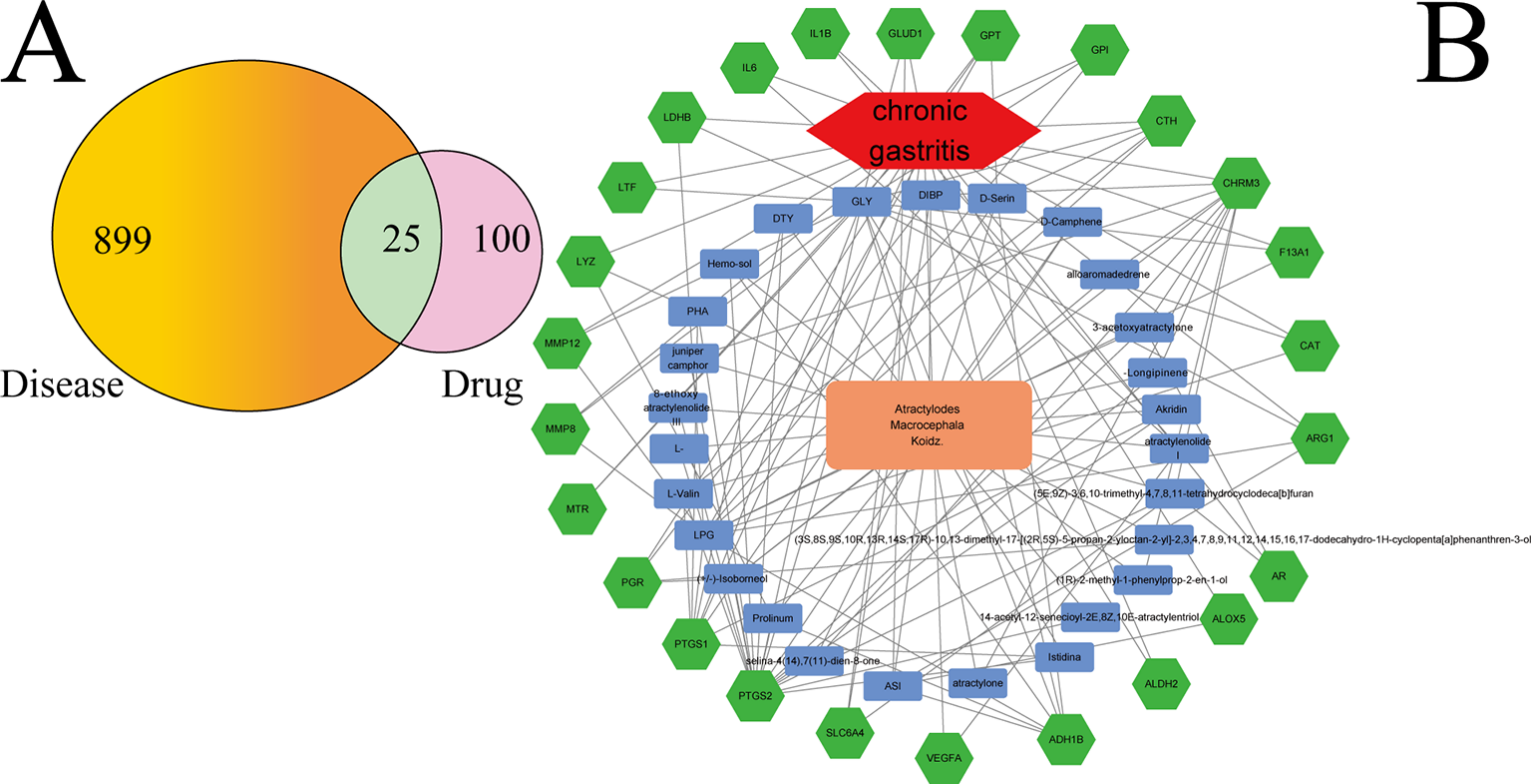
**(A)** Twenty-five overlapping gene symbols between the disease and drug. **(B)** D-I-G-D network. The orange node represents AMK and the red node represents CG. The 27 blue nodes represent the active ingredients in AMK; The 25 green nodes represent the overlapping gene symbols between the disease and drug. The edges denote that nodes can interact with each other.

**Figure 2A** shows that 899 gene symbols for disease and 100 gene symbols for drugs had 25 overlaps. That is, 25 gene symbols may be the key for CG treatment by AMK. The 25 overlapping gene symbols are detailed in **Supplementary Table S4**.

To clarify how AMK may act against CG, we used Ctyoscape to build a D–I–G–D network (**Figure 2B**); the orange node represents AMK and the red node represents CG. Also, the 27 blue nodes represent the active ingredients in AMK; the 25 green nodes represent the overlapping gene symbols between the disease and drug. The edges denote that nodes can interact with each other.

We conducted further network analyses by evaluating centralization and heterogeneity, which were 0.432 and 1.077, respectively. Hence, some nodes were more concentrated in the network than others. That is, the ingredient−target symbol space tended to veer toward certain ingredients and target symbols. Hence, the network included some ingredients with multiple target symbols, such as glycine (GLY; degree = 17), (L)-Alanine (LPG; degree = 12), (5E,9Z)-3,6,10-trimethyl-4,7,8,11-tetrahydrocyclodeca[b]furan (5), 3β-acetoxyatractylone (4), ATL-I (4), and atractylone (2). Moreover, it meant that AMK could act on multiple targets through the same active ingredient. For example, ATL-I could have an effect on vascular endothelial growth factor A (VEGFA), IL-6, and IL-1β if used for CG treatment. Wang and colleagues showed that ATL-I can achieve anti-inflammatory effects by inhibiting expression of IL-6 and IL-1β (Wang et al., 2009). Detailed information regarding active ingredients and gene symbols are described in **Supplementary Table S5**.

### Analyses of a PPI Network

**Figure 3:**
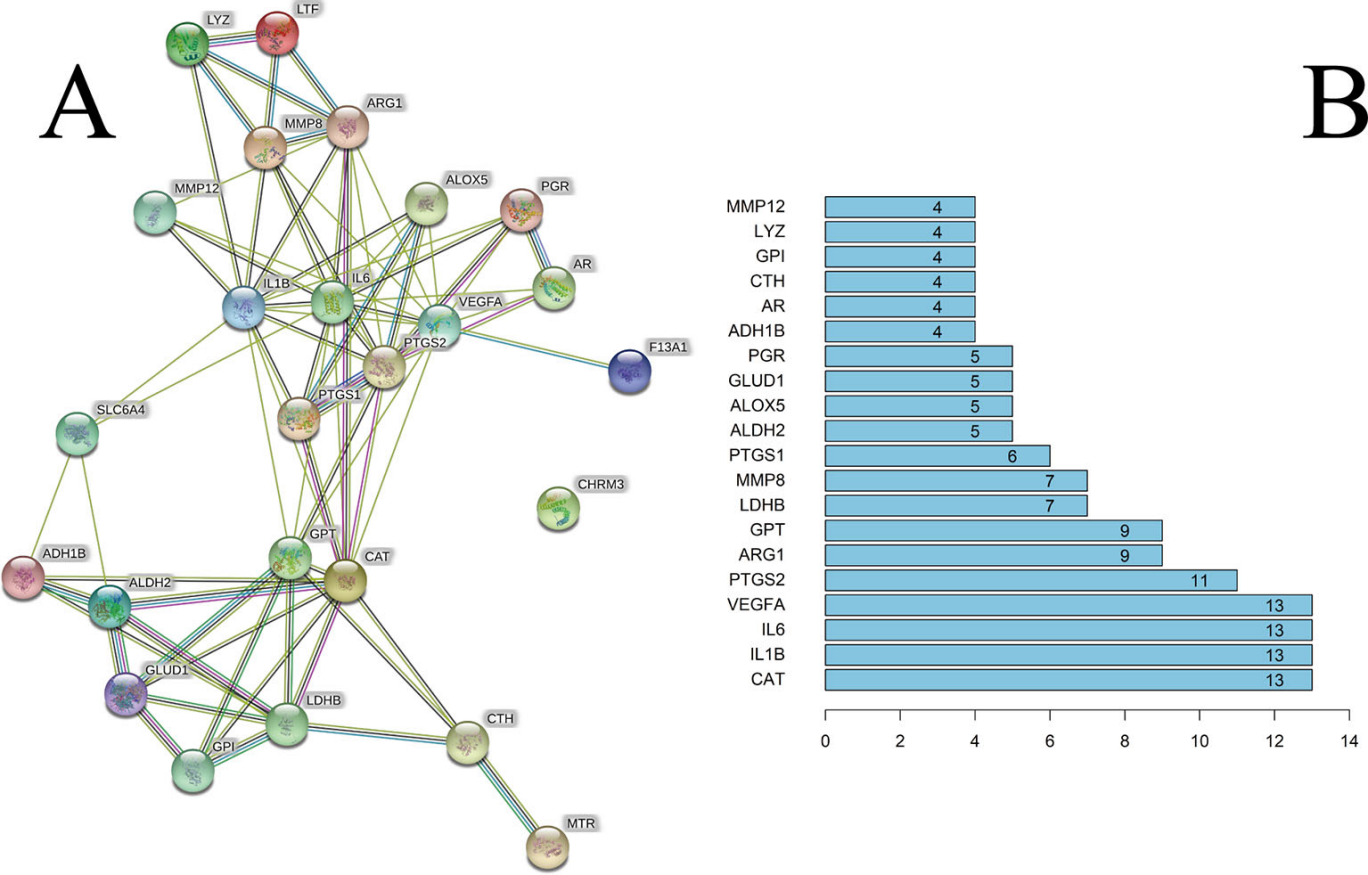
**(A)** The PPI network. **(B)** The bar plot of the PPI network. The x-axis represents the number of neighboring proteins of the target protein. The y-axis represents the target protein.

We constructed a PPI network consisting of 25 nodes and 77 edges (**Figure 3A**). This was based on the premise that proteins have more interactions among themselves than would be expected for a random set of proteins of similar size, drawn from the genome. Such an enrichment indicates that the proteins are at least partially biologically connected, as a group.

The light-blue edges denote known interactions from curated databases. The pink edges denote that the known interactions were determined by experimental methods. The green edges denote that the predicted interactions arose from the neighborhood gene. The red edges denote that the predicted interactions arose from gene fusions. The dark-blue edges denote that the predicted interactions arose from gene co-occurrence. The yellow edges denote that the predicted interactions arose from text-mining. The black edges denote that the predicted interactions arose from co-expression. The lavender edges denote that the predicted interactions arose from protein homology. The details of the PPI network are described in **Supplementary Table S6**.

We took the first 20 proteins in the PPI network. As seen from **Figure 3B**, catalase (CAT), IL-6, IL-1β, and VEGFA could be related to the other 13 proteins. These results suggested that these four proteins would be the focus of our research of PPIs. Prostaglandin G/H synthase 2 (PTGS2) could be related to the other 11 proteins. CHRM3 was not associated with other proteins in this PPI network.

### Analyses of Enrichment of GO Pathways

**Figure 4:**
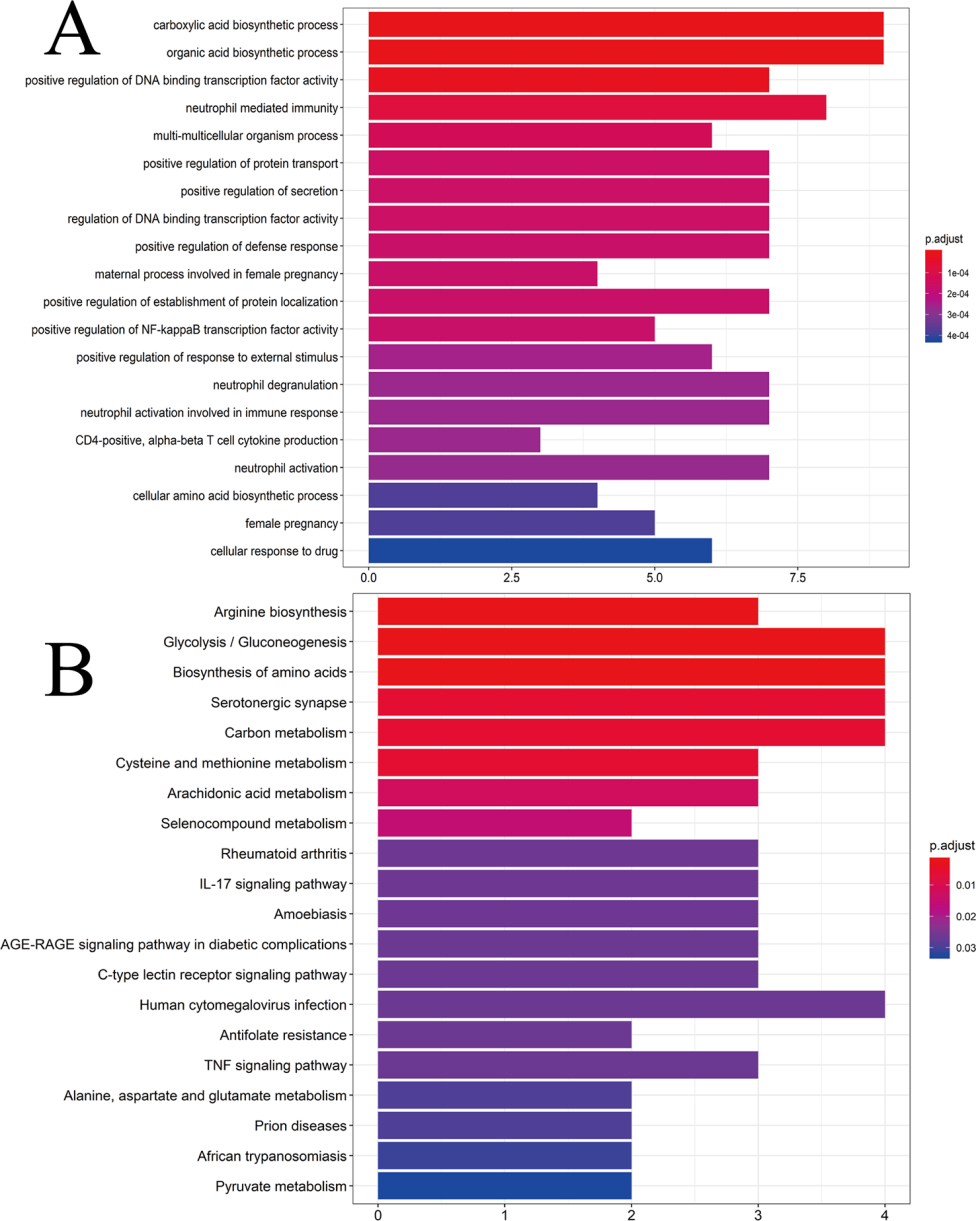
**(A)** GO analyses of the 25 gene symbols associated with chronic gastritis. The x-axis represents significant enrichment in the counts of these terms. The y-axis represents the categories of “biological process” in the GO of the target genes (*p* < 0.01). **(B)** KEGG pathway enrichment analyses. The x-axis represents the counts of the target symbols in each pathway; the y-axis represents the main pathways (*p* < 0.01).

To ascertain if the 25 gene symbols were related to CG, we conducted analyses of enrichment of GO pathways to clarify the relevant biologic processes (*p* < 0.01) (**Figure 4A**). The y-axis represents GO terms. The x-axis indicates the number of genes enriched in that term. The redder the color, the smaller the value of p.adjust (FDR); it also denotes greater credibility and greater importance. In contrast, the bluer the color, the greater is the value of p.adjust. For a brief demonstration, we intercepted the first 20 terms from small to large according to the p-value. The details of GO analyses are described in **Supplementary Table S7**. The results indicated that numerous biologic processes were involved in CG treatment, including carboxylic acid biosynthetic process (GO:0046394), organic acid biosynthetic process (GO:0016053), positive regulation of DNA binding transcription factor activity (GO:0051091), positive regulation of the defense response (GO:0031349), and neutrophil activation involved in the immune response (GO:0002283).

### Analyses of Enrichment of the KEGG Pathway

Analyses of enrichment of the KEGG pathway were carried out using Bioconductor (R) (*p* < 0.01) (**Figure 4B**). The y-axis represents the KEGG pathway. The x-axis indicates the number of genes enriched in that pathway. The redder the color, the smaller the value of p.adjust; it also denotes greater credibility and greater importance. In contrast, the bluer the color, the greater the value of p.adjust. The 25 overlapping gene symbols were mapped to 26 pathways after enrichment of the KEGG pathway. For a brief demonstration, we intercepted the first 20 pathways from small to large according to the p-value. The details of enrichment of the KEGG pathway are described in **Supplementary Table S8**.

Enrichment of the KEGG pathway could be divided approximately into modules of amino acid synthesis, energy metabolism, and inflammation. As shown in **Figure 4B**, the 25 overlapping gene symbols interacted closely with the pathways involved in the IL-17 signaling pathway (hsa04657), C-type lectin receptor signaling pathway (hsa04625), tumor necrosis factor (TNF) signaling pathway (hsa04668), and AGE-RAGE signaling pathway in diabetic complications (hsa04933). These pathways may be the key pathways responsible for CG treatment. Such analyses offer a new way to study CG treatment.

### Computational Validation of Selected Ingredients–Targets Interactions

In general, the number and strength of a ligand bound to a receptor is determined largely by the inhibitory efficiency (Wang et al., 2017). Therefore, we explored the interactions and binding modes between the inflammatory factors IL-6, IL-1β, and gastrointestinal regulatory target CHRM3 with their active ingredients by molecular docking.

**Figure 5:**
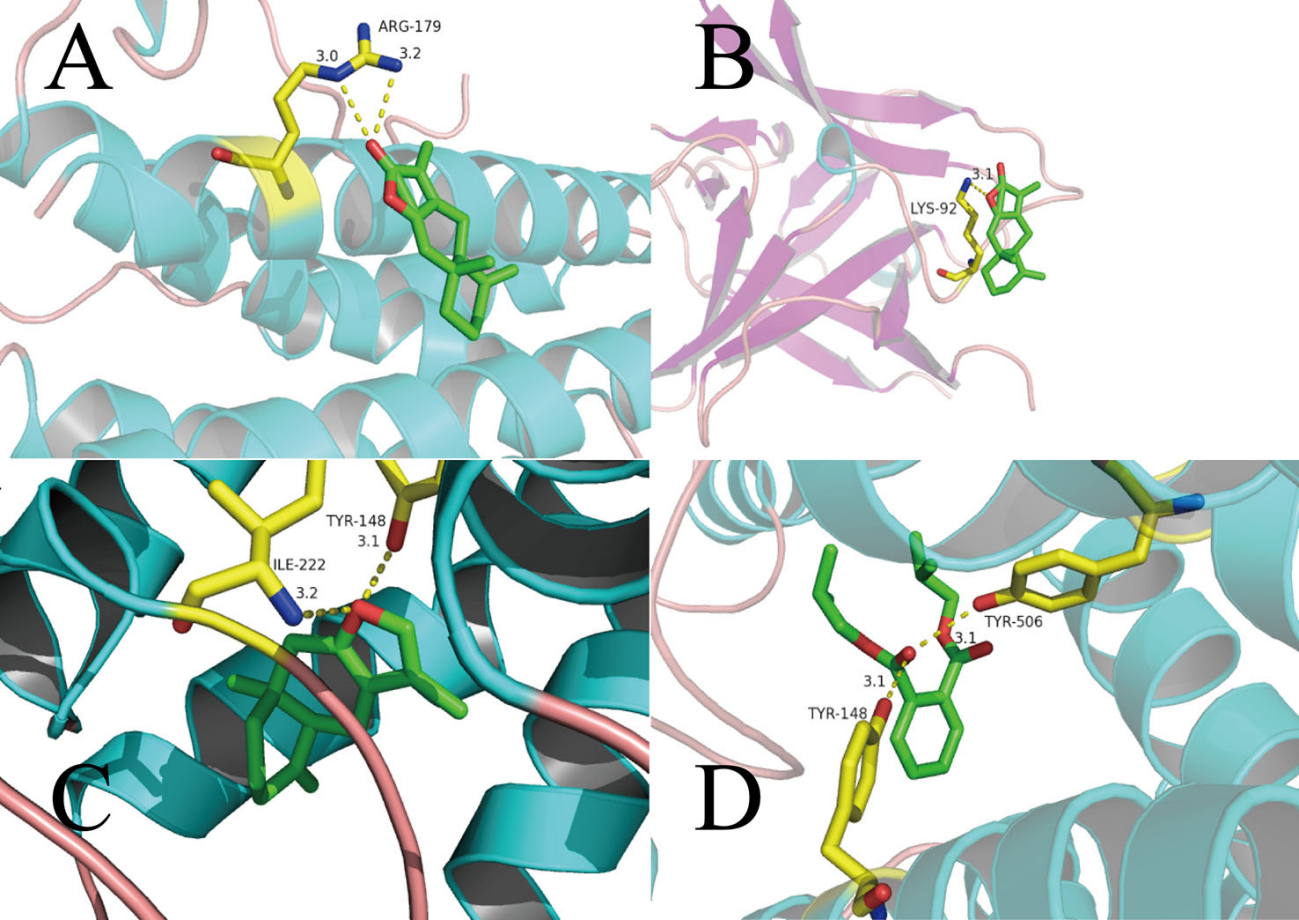
Binding studies of selected ingredients–targets interactions. **(A)** ATL-I with IL-6; **(B)** ATL-I with IL-1β; **(C)** atractylone with CHRM3; **(D)** DIBP with CHRM3. Molecules are represented by a ball-and-stick model, the hydrogen bonds are represented by a dotted line, and the distance is in angstroms. Atoms C, O, and N are green, red, and blue, respectively.

ATL-I has special pharmacologic effects and high content in AMK. Hence, we first conducted molecular docking of ATL-I with IL-6 (**Figure 5A**). We can see clearly from **Figure 5A** that the O atoms in ATL-I and arginine (ARG)-179 (3.0 Å) and ARG-179 (3.2 Å) interact with each other between each N atom. In **Figure 5B**, the molecular-docking result of ATL-I with IL-1β shows that the O atoms on the lactone ring in ATL-I can interact with the N atoms on lysine (LYS)-92 (3.1Å) of IL-1β. The results of molecular docking were also consistent with our cell-experiment results, which demonstrated that ATL-I had a significant anti-inflammatory effect.

As another active ingredient with high content in AMK, atractylone was docked with CHRM3 (**Figure 5C**). The O atoms in atractylone interact with N and O atoms in isoleucine (ILE)-222 (3.2Å) and tyrosine (TYR)-148 (3.1Å), respectively, in CHRM3. This interaction also ensures a tight fit between atractylone and CHRM3. In **Figure 5D**, the carbonyl group in bis(2-methylpropyl) benzene-1,2-dicarboxylate (DIBP) is associated with the O atoms in TYR-148 (3.1Å) and TYR-506 (3.1Å) in CHRM3.

Based on these data, we can consider that the interaction between these active ingredients and targets is the basis of their biologic activity. It also means that AMK has multiple ingredients and multiple targets.

### Experimental Validation *In Vitro*

#### CCK-8 Assay

**Figure 6:**
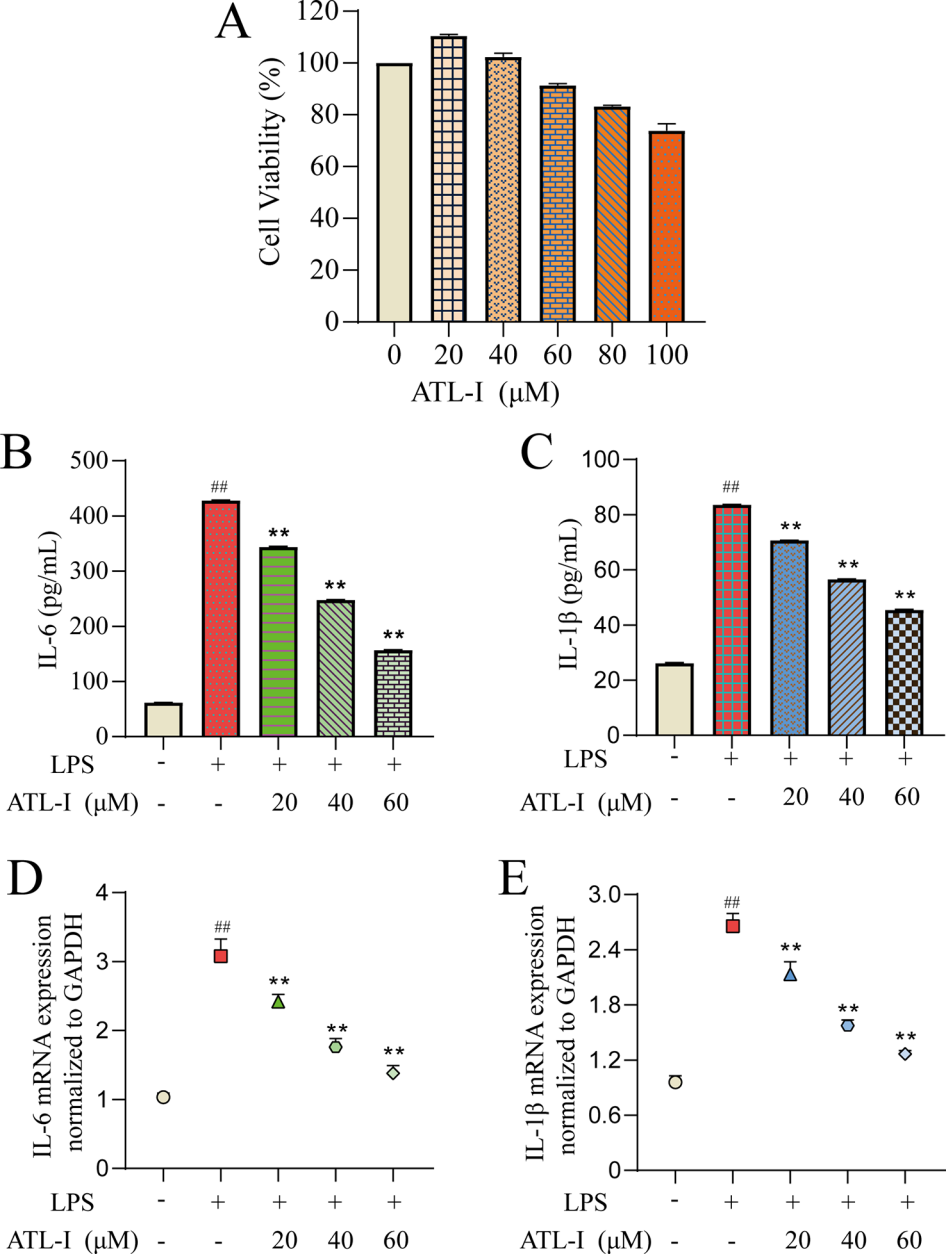
Action of ATL-I on RAW264.7 cells. RAW264.7 cells were incubated with LPS (1 μg/mL) for 24 h and then treated with ATL-I (20, 40, or 60 μM) for 24 h. The effects of ATL-I **(A)** on the viability of RAW264.7 cells using the CCK-8 assay. Production of IL-6 **(B)** and IL-1β **(C)** was determined by ELISAs. Protein expression of IL-6 **(D)** and IL-1β **(E)** was determined by qRT-PCR. ##*p* < 0.01 *versus* blank control group. **p < 0.01 LPS-treated group.

First, we determined the effects of different doses of ATL-I on the viability of RAW264.7 cells using the CCK-8 assay (**Figure 6A**). ATL-I at <60 μmol/L had high cell viability (>90%). Therefore, three concentrations were selected (20, 40, 60 μmol/L) for subsequent experiments.

#### Validation of Targets

To further evaluate the results obtained in systematic pharmacologic analyses, ATL-I was selected from AMK to examine potential anti-inflammatory effects using LPS (1 μg/mL)-stimulated RAW264.7 cells. We undertook ELISAs and qRT-PCR for IL-6 and IL-1β to confirm the predicted anti-inflammatory effects of the ingredients. IL-6 production showed a significant decreasing trend with increasing ATL-I concentration (**Figure 6B**). Simultaneously, a significant decrease in IL-6 mRNA expression was observed (**Figure 6C**). Production and mRNA expression of IL-1β also decreased significantly with increasing ATL-I concentration (**Figures 6D, E**).

To summarize, these data suggested that ATL-I from AMK may have a therapeutic effect against CG because it can regulate expression of IL-6 and IL-1β to inhibit inflammation. *In vitro* studies also provided additional information for screening ingredients with potential anti-inflammatory effects, and demonstrated the rationality of molecular-docking results and the reliability of a screening strategy based on systems pharmacology.

## Discussion

Western medicine often uses antibacterial therapy and gastric-mucosa protection for CG. However, this strategy can lead to bacterial resistance and, increasingly, drugs cannot control CG efficaciously. Some scholars have suggested that TCM can achieve similar (or even better) curative effects than those of western medicine (Qian et al., 2007; Niu and Meng, 2013; Jia et al., 2017). A detailed study showed that traditional Chinese formulations, which include AMK, have biologic effects in patients with gastric diseases of spleen-deficiency and Qi-stagnation syndrome (Zhang et al., 2013). However, the specific pharmacologic mechanism is incompletely understood. Therefore, we explored the mechanism of action of AMK in CG treatment. We applied network pharmacology combined with screening of active ingredients, drug target symbols, and network/pathway analyses.

We showed that 27 active ingredients in AMK influence 25 overlapping gene symbols that have important roles in CG treatment. Thirty GO terms and 26 pathways were obtained by analyses of enrichment of GO pathways and KEGG pathways. We postulate that the IL-17 signaling pathway (hsa04657), C-type lectin receptor signaling pathway (hsa04625), TNF signaling pathway (hsa04668), and AGE-RAGE signaling pathway in diabetic complications (hsa04933) might serve as the key points and principal signaling pathways in CG treatment.

ATL-I and atractylone have been identified as bioactive ingredients by researchers (Chen, 1987; Hwang et al., 1996; Wang et al., 2006). In addition, in TCM theory, AMK is considered to replenish QI. Therefore, when we screened the active ingredients of AMK, although the DL of some amino acids was <0.18 (e.g., GLY, LPG), they were not removed. Our hypothesis was confirmed in analyses of enrichment of GO pathways and KEGG pathways. For example, ingredients such as GLY and LPG can affect alanine aminotransferase 1 (GPT), but GPT is enriched in carbon metabolism, arginine biosynthesis, glycolysis/gluconeogenesis, biosynthesis of amino acids, alanine, aspartate, and glutamate metabolism. Hence, AMK can act through synthetic or metabolic pathways involved in CG treatment.

In addition, the same target can be linked to multiple active ingredients, for example, PTGS2 can be associated with D-camphene, α-longipinene, akridin, and 14-acetyl-12-senecioyl -2E,8Z, 10E-atractylentriol. These data suggest that AMK can act on the same target through multiple active ingredients. ATL-I can be associated with IL-6, IL-1β, and VEGFA, which also suggests that AMK can act on multiple targets through the same active ingredient.

Analyses of a PPI network showed that CAT, IL-6, IL-1β, and VEGFA were the most correlated proteins, followed by PTGS2. In combination with analyses of enrichment of GO pathways and KEGG pathways, we believe that the mechanism of action of AMK in CG treatment is closely related to inflammation regulation (Graham et al., 2000; Bartchewsky et al., 2009; Chen et al., 2018). For example, the target symbol PTGS2 (also named cyclo-oxygenase (COX)-2) can be associated with 18 active ingredients in AMK which can affect the IL-17 signaling pathway, TNF signaling pathway, and C-type lectin receptor signaling pathway and, thus, the regulation of inflammation. If not treated in a timely manner, CG can transform into gastric cancer. Studies have shown that COX-2 plays an important part in the pathogenesis of gastric cancer (Sheu et al., 2003; Bartchewsky et al., 2009). In addition, Zhang and colleagues showed that a reduction in the COX-2 level is related to regression of precancerous lesions (Zhang et al., 2015).

To some extent, the data mentioned above also suggest that AMK has multiple ingredients, multiple targets, and multiple approaches. Such data provide the basis for multi-ingredient synergies in future research.

To summary, network pharmacology has been used widely in TCM research (Yang et al., 2017; Wang et al., 2017).

In China, AMK can be used to treat constipation, irritable bowel syndrome (IBS) and diarrhea (Pan et al., 2009; Xiao et al., 2015; Li and Li, 2015). In TCM theory, patients with spleen deficiency and dampness can suffer diarrhea after taking AMK. The interactions between the herbs in AMK merit investigation. Studies have shown that solute carrier family 6 member 4 (SLC6A4) can have an effect on IBS (Kim et al., 2004; Yuan et al., 2014). The target is associated with diarrhea and constipation. Therefore, we speculate that AMK may have side effects by affecting SLC6A4. But more proof is needed to verify this hypothesis.

We explored, systematically, how AMK may operate in terms of CG treatment. Our data may offer insights into how TCM can be employed in CG treatment.

## Data Availability Statement

The data used to support the findings of our study are included within the article, or within the **Supplementary Material**.

## Author Contributions

SY and HY conceived and designed the study. YY conceived and designed the experimental validation in vitro. All authors participated in drafting of the manuscript and revising it before final submission. These authors: SY, JZ and YY have contributed equally to this work and share first authorship.

## Funding

This work was supported by the National Key Research and Development Project of China (2018YFC1707206).

## Conflict of Interest

The authors declare that the research was conducted in the absence of any commercial or financial relationships that could be construed as a potential conflict of interest.

## Abbreviations

ADME, absorption, distribution, metabolism, and excretion; AMK, *Atractylodes macrocephala* Koidz.; ATL-I, atractylenolide-I; CG, chronic gastritis; CHRM3, muscarinic acetylcholine receptor M3; DL, drug-likeness; GO, Gene Ontology; IL, interleukin; KEGG, Kyoto Encyclopedia of Genes and Genomes; LPS, lipopolysaccharide; OB, oral bioavailability; OMIM, Online Mendelian Inheritance in Man; PPI, protein–protein interaction; PTGS2, prostaglandin G/H synthase 2; STRING, Search Tool for the Retrieval of Interacting Genes/Proteins; TCM, traditional Chinese medicine; TCMSP, Traditional Chinese Medicine Systems Pharmacology Database.
